# A case of phace syndrome and acquired hypopituitarism?

**DOI:** 10.1186/1687-9856-2012-20

**Published:** 2012-06-30

**Authors:** Friederike Denzer, Christian Denzer, Belinda S Lennerz, Harald Bode, Martin Wabitsch

**Affiliations:** 1 Department of Pediatrics and Adolescent Medicine, Division of Pediatric Endocrinology and Diabetes, University Hospital of Ulm, Eythstr. 24, Ulm, D-89075, Germany; 2 Pediatric Neurology Department, University Hospital of Ulm, Eythstr. 24, Ulm, D-89075, Germany; 3 Department of Pediatrics and Adolescent Medicine, Division of Pediatric Endocrinology and Diabetes, University Hospital of Ulm, Eythstr. 24, Ulm, D-89075, Germany

**Keywords:** PHACE syndrome, Hypopituitarism, Growth hormone deficiency, Central hypothyroidism, Neurocutaneous syndrome

## Abstract

**Background:**

PHACE is a neurocutaneous syndrome associated with: Posterior fossa brain malformations, large “segmental” facial hemangiomas, arterial cerebrovascular-, cardiovascular-, and eye anomalies.

**Case vignette:**

We are reporting a girl with PHACE syndrome. The patient had a congenital right-sided facial hemangioma with plaque-morphology. At age 11 years and 2 months she presented with short stature, markedly decreased growth velocity and signs and symptoms suggestive of hypothyroidism. Magnetic Resonance Imaging (MRI) of the brain revealed complex structural and cerebrovascular arterial anomalies, including an empty sella. Testing of pituitary function revealed multiple pituitary dysfunctions, including absolute growth hormone deficiency, hypogonadotropic hypogonadism, central hypothyroidism, and secondary adrenal insufficiency.

**Conclusions:**

This case suggests the necessity to screen all patients with PHACE syndrome and intracranial malformations for pituitary dysfunction at regular intervals.

## Background

PHACE syndrome (OMIM 606519) is a neurocutaneous syndrome first described by Frieden et al. in 1996 [1]. PHACE syndrome refers to the association of a large, segmental hemangioma, associated with one or more of the following features: posterior fossa brain malformations (e.g. Dandy-Walker-complex, cerebellar hypoplasia or atrophy, and dysgenesis or agenesis of vermis), arterial cerebrovascular anomalies, cardiovascular anomalies, and eye anomalies. The etiology and pathogenesis are unknown. Potential comorbidities are mainly complications of cerebral and cerebrovascular anomalies and include seizures and ischemic strokes [2,3]. To date, a total of approximately 300 patients with PHACE syndrome are reported in the literature. Contributions range from case reports and smaller case series with e.g 25 [2] and 12 [4] patients, to larger series with 130 [3], 70 [5] and 43[1] patients respectively.

To date, a total of three patients with PHACE syndrome and congenital pituitary dysfunctions have been described. Poindexter et al. reported a patient with congenital panhypopituitarism and cerebral and cardiovascular anomalies diagnosed at age 13 months [6]. Metry et al. reported a patient with ocular coloboma, partial empty sella, and isolated growth hormone (GH) deficiency [2]. Merheb at al reported a 16 year old patient with central hypothyroidism and growth hormone deficiency [7]. Hypogonadotropic hypogonadism was suspected but not confirmed.

Here we report a case of PHACE syndrome and hypopituitarism that appears to be acquired, an association that is not previously described.

## Case presentation

**Figure 1:**
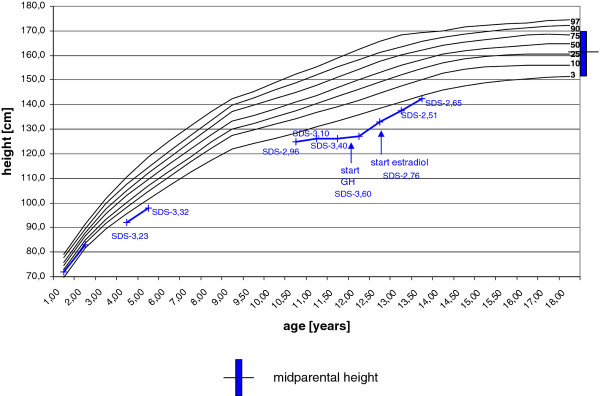
**Patient’s Growth Chart.** Measured height of the patient before and after starting GH substitution is plotted on reference height percentiles according to Prader et al., 1989 [8].

The 11 years and 2 months old girl presented to the pediatric endocrinology department for evaluation of short stature . After normal longitudinal growth in infancy, the patient’s height SDS dropped from −1,67 SDS to −3.23 SDS between the ages of 2.5 and 4.5 years. Subsequently, she resumed normal height velocity, and height SDS at 10.5 years was preserved at −2.96. At presentation, height SDS was −3.10, weight SDS −2.38 (Figure 1) and documented height velocity was SDS −2.2. The midparental height SDS was 0.59.

Central hypothyroidism had been diagnosed 4 months before presentation. The girl is of normal intelligence, her school achievement is above average.

As far as we can determine retrospectively, there was no clinical suspicion of hypoglycemia during the newborn period. There was no history of severe illness. Febrile infections were tolerated well and could all be treated at home.

**Figure 2:**
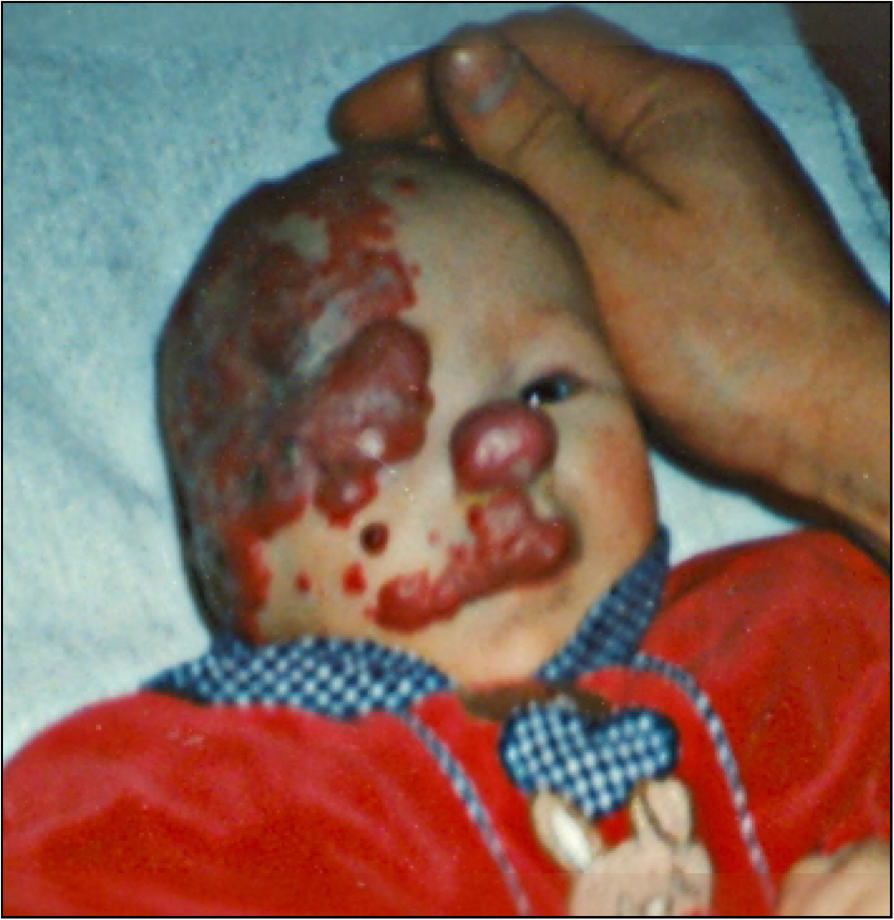
**Picture of the Patient.** Photography of the patient in infancy before treatment of the haemangioma. At this stage, PHACE syndrome could already have been suspected due to the typical finding of a segmental hemangioma.

The patient had a history of a congenital right-sided facial hemangioma of plaque-morphology (Figure 2). This was treated in infancy with interferone- followed by laser therapy. The clinical examination revealed a prepubertal stage (Tanner PH1, B1).

### **Laboratory studies**

Because of the described empty sella on MRI, comprehensive endocrine function testing was performed. Reference ranges are given in parentheses.

· The GnRH test showed central hypogonadism (Table 1). Estradiol was undetectable (<5 ng/l) until treatment with estradiolvalerate was initiated.

**Table 1:** Results of endocrine function testing

			**0 min**	**30 min**	**45 min**	**60 min**	**120 min**
GnRH test	LH	IU/l	< 0.1		<0.1		
	FSH	IU/l	0.24		0.54		
arginine test 1	HGH	μg/l	0.07	<0.05		0.05	<0.05
arginine test 2	HGH	μg/l	0,09	0,13		0,13	<0,05

A GnRH test was performed by administering 3.5 μg/kg GnRH by intravenous injection. Blood samples were taken at baseline and 45 minutes after GnRH application. Two independent arginine tests were performed by applying l-argininehydrochloride 21 % 0.5 g /kg. Blood samples were taken at start and after 30, 60 and 120 minutes.

· A diurnal profile of cortisol incretion was not obtainable, but repeated measurements of ACTH and serum cortisol after an overnight fast showed persistently low levels for both parameters, with a peak cortisol level of 2.7 μg/dl (6.2-19.4), and a peak ACTH level of 12 pg/ml (7.2-63.3). 24 h urinary cortisol excretion was low with 4,4 μg/d (36.0-137.0), and DHEAS was low with 18.1 μg/dl (33.9-280.0).

· Thyroid stimulating hormone (TSH) levels never exceeded 0.024 mIU/l (reference range 0.51-4.3), supporting the diagnosis of central hypothyroidism. Free thyroid hormone (fT4) levels were normal on levothyroxine supplementation.

· Arginine stimulation test, performed twice on separate days, revealed absolute growth hormone deficiency (see Table 1). IGF-1 and IGFBP-3 were normal initially, but decreased before starting GH therapy 9 months after the initial presentation to 25 ng/ml (122–155) and 2460 ng/ml (2600–8100) respectively.

In summary, our patient suffered from hypogonadotropic hypogonadism, secondary adrenal insufficiency, central hypothyroidism and absolute growth hormone deficiency.

### **Imaging studies**

**Figure 3:**
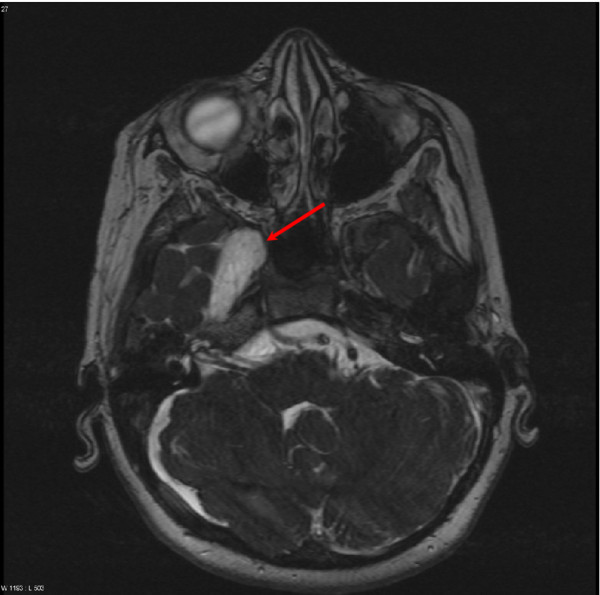
**Arachnoidal cysts.** MRI Scan of the brain. Coronar, T2-weighted. Cerebellar dysplasia is seen on the right side. The arrow indicates a large arachnoidal cyst.

**Figure 4:**
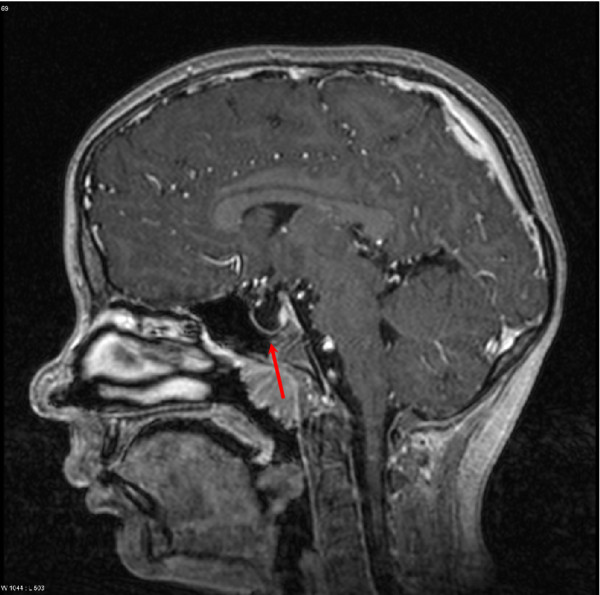
**Empty sella.** MRI Scan of the brain. Sagittal, T2-weighted. The arrow indicates the empty sella.

MRI of the brain confirmed the findings of complex structural cerebral- and cerebrovascular arterial anomalies with cerebellar dysplasia on the right side, deformation and dislocation of the brain stem, and aplasia of the right carotid (Figure 3). The empty sella (Figure 4) appeared to be caused by dorsal displacement of the infundibulum by arachnoidal cysts, which were stable in size over the course of one year. Cranial of the neurohypophysis a structure that appears to be a considerably reduced adenohypophysis was identified.

To rule out further organ involvement, an echocardiography and electrocardiography, as well as an abdominal ultrasound were performed and were unremarkable.

The radiologic bone age of the left hand was 4 years delayed according to the standards of Greulich and Pyle.

### **Treatment**

We initiated substitution of hydrocortisone at 10 mg/m2/d p.o. immediately and GH treatment with 25 μg/kg/d s.c. after 9 months (Patient aged 11 years and 11 months). The delay in starting GH treatment was due to the patient's intense fear of injections and therefore reluctance of the family to start treatment. In the absence of spontaneous pubertal development, Estradiolvalerate 0,3 mg/d was started at the age of 13 years and increased to 0,6 mg/d after 6 months at pubertal stage B2, PH2. The treatment with thyroxine was continued.

The patient is now 13 years and 6 months old. Her height has reached the 3. Percentile (−2,5 SDS)(Figure 1) and stage of puberty is Tanner B2, PH2.

## Conclusions

Hypogonadotropic hypogonadism, secondary adrenal insufficiency, central hypothyroidism and absolute growth hormone deficiency diagnosed at the age of 11 years led to a comprehensive diagnostic work-up revealing the diagnosis of PHACE syndrome. While acquired hypopituitarism has not yet been reported in association with PHACE syndrome, there are several factors that make *congenital* hypopituitarism unlikely in our patient:

The patient had normal growth velocity from 4.5 to 10 years of life. Thus, while there may have been some degree of growth hormone insufficiency early on, absolute congenital growth hormone deficiency seems unlikely. Further evidence of intact growth hormone- and adrenal axes in early childhood includes the absence of symptomatic hypoglycemia during the newborn period, the absence of episodes of severe illness, and the ability to tolerate febrile infections well with no need for hospitalizations. Congenital hypothyroidism can essentially be ruled out by the patient’s normal intelligence and academic achievement.

One possible explanation for the acquired hypopituitarism is that the arachnoidal cyst located in the area of the sella may have caused chronic pressure on the adenohypophysis, leading to a progressive loss of function. As reported in the literature, intracranial cysts can cause a wide spectrum of endocrine insufficiencies [9]. Another potential mechanism causing a more acute onset of pituitary dysfunctions might have been a thromboembolic event with infarction of the adenohypophysis. However, no case of endocrine dysfunction after a thrombembolic event has been reported in the literature, and the patient has no clinical correlates and no MRI evidence of a thrombembolic event.

We conclude that screening of pituitary functions in patients with PHACE syndrome and intracranial malformations is recommendable both at diagnosis and during follow up.

We suggest yearly follow up including: Anthropometric measurements, clinical assessment of pubertal development, and laboratory evaluations of TSH, fT4, ACTH and Cortisol. Decreased height velocity or delayed puberty should warrant further diagnostic evaluation.

## Consent

Written informed consent was obtained from the patient for publication of this Case report and the accompanying images. A copy of the written consent is available for review by the Editor-in-Chief of this journal.

## Competing interests

The authors declare that they have no competing interests.

## Authors’ contributions

FD diagnosed the patient and drafted the manuscript. CD revised the manuscript. BL revised the manuscript. HB made substantial contributions to the process of diagnosing the patient. MW has revised the manuscript and gave final approval of the version to be published. All authors read and approved the final manuscript.
